# Nonlinear-Model-Based Analysis Methods for Time-Course Gene Expression Data

**DOI:** 10.1155/2014/313747

**Published:** 2014-01-02

**Authors:** Li-Ping Tian, Li-Zhi Liu, Fang-Xiang Wu

**Affiliations:** ^1^School of Information, Beijing Wuzi University, No. 1 Fuhe Street, Tongzhou District, Beijing 101149, China; ^2^Department of Mechanical Engineering, University of Saskatchewan, 57 Campus Drive, Saskatoon, SK, Canada S7N 5A9; ^3^Division of Biomedical Engineering, University of Saskatchewan, 57 Campus Drive, Saskatoon, SK, Canada S7N 5A9

## Abstract

Microarray technology has produced a huge body of time-course gene expression data and will continue to produce more. Such gene expression data has been proved useful in genomic disease diagnosis and drug design. The challenge is how to uncover useful information from such data by proper analysis methods such as significance analysis and clustering analysis. Many statistic-based significance analysis methods and distance/correlation-based clustering analysis methods have been applied to time-course expression data. However, these techniques are unable to account for the dynamics of such data. It is the dynamics that characterizes such data and that should be considered in analysis of such data. In this paper, we employ a nonlinear model to analyse time-course gene expression data. We firstly develop an efficient method for estimating the parameters in the nonlinear model. Then we utilize this model to perform the significance analysis of individually differentially expressed genes and clustering analysis of a set of gene expression profiles. The verification with two synthetic datasets shows that our developed significance analysis method and cluster analysis method outperform some existing methods. The application to one real-life biological dataset illustrates that the analysis results of our developed methods are in agreement with the existing results.

## 1. Background 

To understand the mechanisms of dynamic biological processes, DNA microarray experiments have been employed to produce gene expression profiles at a series of time points, for example, the cell division cycle processes of yeast *Saccharomyces cerevisiae* [1, 2], bacterium *Caulobacter crescentus* [3], and human being [4]. Such time-course gene expression data provides a dynamic snapshot of most (if not all) of the genes related to the biological development process and thus can be useful in genomic disease diagnosis and genomic drug design. The challenge is how to uncover useful information from such data by proper analysis methods [5].

Although the behaviours of genome-wide genes can be monitored simultaneously with the current DNA microarray technology, not are all of monitored genes closely related to the biological process being studied or of interest. In addition, gene expression data are often contaminated by various noises or noisy genes. It is impossible to uncover some useful information without any preprocessing. Either excluding genes of interest or including noisy genes could degrade the significance of any analysis results. Therefore, it is critical to select the genes which are closely relevant to a biological process from gene expression profiles measured during the biological process. The selection of genes can be performed by the so-called significance analysis of gene expression profiles. Much attention has been paid to the significant analysis of static gene expression data over the past years. For gene expression data obtained from a pair of conditions (e.g., normal versus abnormal, or control versus treatment) with multiple replicates, one of the widely used approaches in early years is called the *R*-fold change method [6, 7]. The “*R*-fold change” method determines a gene to be significantly expressed if the ratio of expression values under two different conditions is greater than *R* or less than 1/*R*, where *R* is a user-preset positive number. This approach has been improved by a resampling (bootstrapping) method called SAM [8, 9]. Another approach to the significance analysis is the use of *t*-test, for example, on logarithm of the expression levels. In a *t*-test, the means and variances of gene expressions from a pair of conditions are used to compute a normalized distance so-called *t*-value. When the *t*-value exceeds a certain threshold depending on the confidence level selected, gene expression data from a pair of conditions are considered to be significantly different. Although *R*-fold and *t*-test approaches can be extended to apply for the analysis of gene expression data with multiple conditions, for example, SAM [8, 9] and RIT [10], these approaches need the assumption that multiconditional values are statistically independent. Therefore, it is not applicable to time-course gene expression profiles as they are not statistically independent but dynamically dependent. In recent year, we have developed several methods for significance analysis of time-course gene expression data. In [11, 12], we employ linear regression models to detect the significantly differentially expressed genes. In [13, 14], we employ nonlinear models to detect the periodically expressed genes.

Besides the significance analysis, the cluster analysis is another class of analysis methods to uncover the useful information from gene expression data [5]. A number of clustering methods have been proposed for cluster analysis of gene expression data. These include distance/correlation-based clustering methods (e.g., hierarchical clustering [15], *k*-means clustering [16], and self-organizing maps [17]) and static-model-based clustering methods [18, 19]. In these methods, gene expression profiles are viewed as multidimensional vectors. Distance/correlation-based clustering methods cluster genes based on the distance/correlation among their expression profiles. Static-model-based clustering methods assign genes to one of the clusters if their expression profiles are generated by a multivariate normal distribution. These methods do not take the dynamic of time-course gene expression data into account and thus are not efficient for periodically expressed gene data. Some dynamic-model-based clustering methods have been proposed to analyze time-course gene expression data [20, 21]. These methods employ linear autoregressive models to describe the dynamics of time-course gene expression data. Recently we propose the nonlinear-model-based method for clustering periodically expressed genes [22, 23].

As measured from typical nonlinear biological systems, time-course gene expression profiles should display the nonlinear properties. In this paper, we propose nonlinear-model-based methods for significance analysis and cluster analysis of time-course gene expression data. The proposed nonlinear model can be viewed as a generalization of many existing models [13, 14, 20–23]. A two-step method is proposed to estimate the model parameter. An *F*-test is employed to determine if a gene expression profile is significantly different from noisy data. A relocation-iteration algorithm is employed to assign each gene to an appropriate cluster. A bootstrapping method and an average adjusted Rand index (AARI) are employed to measure the quality of clustering. We employ two synthetic datasets to evaluate the performance of the proposed methods and apply them to one real-life biological dataset.

## 2. Methods

### 2.1. Nonlinear Model for Time-Course Gene Profiles

Let *x*(*t*)  (*t* = 1, 2,…, *m*) be a time-course gene expression profile generated from a dynamic biological process, where *m* is the number of time points at which gene expression is measured. Many nonlinear gene expression profiles contain a periodic component and a long-term decrease or increase component. In this study, we employ the following nonlinear model to describe time-course gene expression data:

(1) $$\begin{array}{c} x\left(t\right)=e^{\alpha t}\left[a\cos\left(\omega t\right)+b\sin\left(\omega t\right)\right]+ct+d+\varepsilon \left(t\right), \end{array}$$

where parameter *α* represents the degradation rate of periodicity; parameters *a* and *b* are the coefficients of sine and cosine functions, respectively; parameter *ω* is the frequency of periodic expression data; parameters *c* and *d* are the coefficients of linear function; and *ε*(*t*) represents random errors. This study assumes that the errors have a normal distribution independent of time with the mean of 0 and the variance of *σ* ^2^. This model generalizes several existing models; for example, setting *α* = *c* = *d* = 0, model (1) is reduced to sinusoidal function model [24–30]:

(2) $$\begin{array}{c} x\left(t\right)=A\sin\left(\omega t+\Phi \right)+\varepsilon \left(t\right), \end{array}$$

which is widely used to generate the synthetic periodic gene expression profiles [24] and to detect the periodically expressed genes [27–29]. In model (2), $A=\sqrt{a^{2}+b^{2}}$ is called the magnitude and Φ = arc tan(*a*/*b*) is called the phase. Setting *α* = 0, model (1) is reduced to a model used in [13], while, setting *c* = *d* = 0, model (1) is reduced to a model used in [14, 22]. As model (1) is the generalization of several existing models, it is expected that the analysis results based on this model are better than those reduced models.

To construct model (1) six parameters need to be estimated from a time-course gene expression profile *x*(*t*)  (*t* = 1, 2, …, *m*). Obviously estimating those parameters in model (1) is a nonlinear estimation problem as *α* and **ω** are nonlinear in the model. In general, all nonlinear optimization programs can be used to estimate parameters in model (1), for example, Gauss-Newton iteration method and its variants such as Box-Kanemasu interpolation method, Levenberg damped least squares methods, and Marquardt's method [31–33]. However, these iteration methods are sensitive to initial values. Another main shortcoming is that these methods may converge to the local minimum of the least squares cost function and thus cannot find the true values of the parameters.

Our observation is that noise free model (1)

(3) $$\begin{array}{c} x\left(t\right)=e^{\alpha t}\left[a\cos\left(\omega t\right)+b\sin\left(\omega t\right)\right]+ct+d \end{array}$$

can be viewed as the general solution of the following second order ordinary differential equation:

(4) $$\begin{array}{c} \ddot{x}\left(t\right)+A\dot{x}\left(t\right)+Bx\left(t\right)=Ct+D, \end{array}$$

which is independent of *a* and *b* and

(5) $$\begin{array}{c} \alpha =-\frac{A}{2},\;\;\omega =\frac{\sqrt{4B-A^{2}}}{2},\\ c=\frac{C}{B},\;\;d=\frac{DB-AC}{B^{2}}. \end{array}$$

Now we can see that constant parameters *A*, *B*, *C*, and *D* are linear in (4). As long as we get the first and second derivatives, we can easily estimate the parameters *A*, *B*, *C*, and *D* by the linear least squares method. Then we can get the estimation of *α*, *ω*, *c*, and *d* from equations in (5). Finally we can use (3) to estimate the rest of parameters *a* and *b*. Therefore, we propose the following two-step parameter estimation methods to estimate all six parameters in model (1).

*Step1.* Numerically calculate the first and second derivatives of *x*(*t*). As time-course gene expression data are discrete, the first and second derivatives of *x*(*t*) can be estimated by the central (second order) finite difference formulas as follows:

(6) $$\begin{array}{c} \dot{x}\left(t\right)=\frac{x\left(t+1\right)-x\left(t-1\right)}{2\Delta }\;\text{for}\;t=2,\ldots ,m-1, \end{array}$$

(7) $$\begin{array}{c} \ddot{x}\left(t\right)=\frac{x\left(t+1\right)+x\left(t-1\right)-2x\left(t\right)}{\Delta^{2}}\;\text{for}\;t=2,\ldots ,m-1, \end{array}$$

respectively, where Δ is the time difference between two consecutive gene expression data points. If the number of data points in a gene expression profile is enough, one can choose a high order finite difference formula to get more accurate estimation of these derivatives.

Then, based on model (4), we use the linear least squares method to estimate parameter *ω* ^2^. In detail, let

(8) $$\begin{array}{c} Y=\left[\begin{array}{c} \ddot{x}\left(1\right)\\ \ddot{x}\left(2\right)\\ \vdots \\ \ddot{x}\left(l\right) \end{array}\right],\;\;X=\left[\begin{array}{cccc} -\dot{x}\left(1\right) & -x\left(1\right) & t_{2} & 1\\ -\dot{x}\left(2\right) & -x\left(2\right) & t_{3} & 1\\ \vdots & \vdots & \vdots & \vdots \\ -\dot{x}\left(l\right) & -x\left(l\right) & t_{m-1} & 1 \end{array}\right]. \end{array}$$

From (6) and (7), we have *l* = *m* − 2. Then by the least squares method, the paramters *A*, *B*, *C*, and *D* in model (4) can be estimated as

(9) $$\begin{array}{c} \left[\begin{array}{c} \hat{A}\\ \hat{B}\\ \hat{C}\\ \hat{D} \end{array}\right]={\left(X^{T}X\right)}^{-1}X^{T}Y. \end{array}$$

Note that if the value of $4\hat{B}-{\hat{A}}^{2}$ calculated by (5) for a gene is negative, the expression of this gene will be judged not to be described by model (1).

*Step2.* Substitute the estimated values of *α*, *ω*, *c*, and *d* into (3). Then we apply the least squares method to model (1) to estimate parameters *a* and *b*. In detail, let

(10) $$\begin{array}{c} Z=\left[z\left(1\right),\ldots ,z\left(m\right)\right],\\ E=\left[\begin{array}{c} \cos\left(\Delta \hat{\omega }\right),\ldots ,\cos\left(m\Delta \hat{\omega }\right)\\ \sin\left(\Delta \hat{\omega }\right),\ldots ,\sin\left(m\Delta \hat{\omega }\right) \end{array}\right]; \end{array}$$

by the least squares method, *a* and *b* can be estimated as

(11) $$\begin{array}{c} \left[\begin{array}{c} \hat{a}\\ \hat{b} \end{array}\right]={\left(EE^{T}\right)}^{-1}\left(EZ^{T}\right), \end{array}$$

where

(12) $$\begin{array}{c} z\left(t\right)=e^{-\hat{\alpha }t}\left[x\left(t\right)-\hat{c}t-\hat{d}\right]\;\text{for}\;\;t=1,2,\ldots ,m. \end{array}$$

### 2.2. Nonlinear-Model-Based Significance Analysis

Significance analysis of gene expression data is to determine if a gene expression profile is significantly different from noisy data. This issue is not easy to answer through statistical inference [29, 30] yet, especially for time-course gene expression profiles as their data points are not statistically independent. However, a practical way in the literature [27–30] is to perform a statistical hypothesis test whether the gene expression profile is pure normal white noise or it fits a certain model as specified by (1). Along with this way, this study tests the null hypothesis of

(*H*_0_) (13) $$\begin{array}{c} x\left(t\right)=d+\varepsilon \left(t\right) \end{array}$$

versus the alternative hypothesis of

(*H*_1_) (see (1)).

Let

(14) $$\begin{array}{c} S_{0}^{2}=\overset{m}{\underset{i=1}{\sum }}{\left(x\left(t_{i}\right)-\hat{d}\right)}^{2},\;\;\hat{d}=\frac{1}{m}\overset{m}{\underset{i=1}{\sum }}x\left(t_{i}\right), \end{array}$$

where *S*_0_^2^ is the residual of model (13) with estimated parameters, and

(15) $$\begin{array}{c} S_{1}^{2}=\overset{m}{\underset{i=1}{\sum }}{\left\{x\left(t_{i}\right)-e^{\hat{\alpha }t}\left[a\cos\left(\hat{\omega }t_{i}\right)+\hat{b}\sin\left(\hat{\omega }t_{i}\right)\right]-\hat{c}t_{i}-\hat{d}\right\}}^{2}, \end{array}$$

where *S*_1_^2^ is the residual of model (1) with estimated parameters. As the noise model (13) can be viewed as a special case of model (1), the statistic

(16) $$\begin{array}{c} F=\frac{\left(S_{0}^{2}-S_{1}^{2}\right)/5}{S_{1}^{2}/\left(m-6\right)}=\frac{m-6}{5}\left(\frac{S_{0}^{2}}{S_{1}^{2}}-1\right) \end{array}$$

follows the *F*-distribution with the degrees of freedom (5, *m* − 6), according to statistics theory [21, 23].

When the value of *F*-statistic is large enough (greater than a specified threshold), model (13) is rejected; that is, the gene expression profile is not pure normal white noise, and otherwise the gene expression profile appears as white noises. According to degrees of freedom (which are related to the length of time-course data *m* and the number of parameters in the models) and a significance level (typically, 0.01, 0.05, 0.1, 0.2, or the like) specified by a user, the threshold value can be determined from *F*-distribution table or by using a *f*-distribution table or a standard MATLAB function *icdf*(“*f*”, 1 − *γ*, 5, *m* − 6), where *γ* is the significance level. A significance level is the probability that the null hypothesis is true. Therefore, the rejection of the null hypothesis at a smaller significance level indicates the more favourable to alternative hypothesis. That is, the smaller the significance level is, the more confidence one accepts that genes are not noises if its corresponding value of *F*-statistic is greater than the threshold.

### 2.3. Nonlinear-Model-Based Cluster Analysis

#### 2.3.1. The Mixture Model

In this study, it is assumed that a time-course gene expression dataset is a collection of time series which belongs to several clusters and time series in each cluster can be described by model (1) with different parameters. Let *θ*_*k*_ = [*α*_*k*_, *ω*_*k*_, *a*_*k*_, *b*_*k*_, *c*_*k*_, *d*_*k*_] be parameters of model (1) for the *k*th cluster. Then the task of nonlinear-model-based clustering is as follows: for a given number of cluster *K*, divide a time-course gene expression dataset into a partition *C* = {*C*_1_,…, *C*_*k*_,…, *C*_*K*_} using model (1) with parameters *θ*_*k*_ = [*α*_*k*_, *ω*_*k*_, *a*_*k*_, *b*_*k*_, *c*_*k*_, *d*_*k*_](*k* = 1,…, *K*) which minimize

(17) f(C ∣ Θ)  =∑k=1K∑x∈Ck∑i=1m{x(i)−eαkΔi[akcos(iΔωk)+bksin(iΔωk)]   −ckΔi−dk}2,

where the parameters Θ consist of {*θ*_*k*_,  *k* = 1,…, *K*}. 

#### 2.3.2. Estimation of Cluster Parameters

According to the parameter estimation method proposed in previous section for single time-course expression profiles, for the *k*th cluster, parameters *θ*_*k*_ = [*α*_*k*_, *ω*_*k*_, *a*_*k*_, *b*_*k*_, *c*_*k*_, *d*_*k*_] can be estimated as

(18) $$\begin{array}{c} {\hat{\alpha }}_{k}=-\frac{{\hat{A}}_{k}}{2},\;\;{\hat{\omega }}_{k}=\frac{\sqrt{4{\hat{B}}_{k}-{\hat{A}}_{k}^{2}}}{2},\\ {\hat{c}}_{k}=\frac{\hat{C}}{\hat{B}},\;\;{\hat{d}}_{k}=\frac{{\hat{D}}_{k}{\hat{B}}_{k}-{\hat{A}}_{k}{\hat{C}}_{k}}{{\hat{B}}_{k}^{2}}, \end{array}$$

where

(19) $$\begin{array}{c} \left[\begin{array}{c} {\hat{A}}_{k}\\ {\hat{B}}_{k}\\ {\hat{C}}_{k}\\ {\hat{D}}_{k} \end{array}\right]={\left(\overset{}{\underset{x\in C_{k}}{\sum }}X^{T}X\right)}^{-1}\overset{}{\underset{x\in C_{k}}{\sum }}X^{T}Y,\\ \left[\begin{array}{c} {\hat{a}}_{k}\\ {\hat{b}}_{k} \end{array}\right]={\left(\sum_{x\in C_{k}}EE^{T}\right)}^{-1}\sum_{x\in C_{k}}EZ^{T}, \end{array}$$

where |*C*_*k*_| represents the number of time series in cluster *C*_*k*_, ∑_*k*=1_^*K*^|*C*_*k*_| = *N*. 

#### 2.3.3. Algorithm for Clustering

This study employs the following relocation-iteration algorithm to estimate the parameters such that the cost function (17) is minimized:

(1) select an initial partition for the given number of clusters, *K*;
(2) iterate (*s* = 1,2,…):
  (a) estimate the parameter Θ^*s*^ based on the current partition by using (18)-(19);
  (b) generate a new partition by assigning each sequence *x* to cluster *k* where
    (20) k=arg min1≤j≤K∑i=1m{x(i)−eαjsΔi[ajscos(iΔωjs)+bjssin(iΔωjs)] − cjsΔi−djs}2;
(3) stop if the improvement of the cost function (17) is below a given threshold, or the cluster memberships of time series do not change significantly.

In 2(a), Θ^*s*^ = {*θ*_*k*_^*s*^, 1 ≤ *k* ≤ *K*} represents the estimated parameters in cost function (17) at iteration *s* while in 2(b), parameters *α*_*j*_^*s*^, *ω*_*j*_^*s*^, *a*_*j*_^*s*^, *b*_*j*_^*s*^, *c*_*j*_^*s*^,   and *d*_*j*_^*s*^ represent the parameters of model *j* at iteration *s*.

## 3. Evaluation

**Figure 1:**
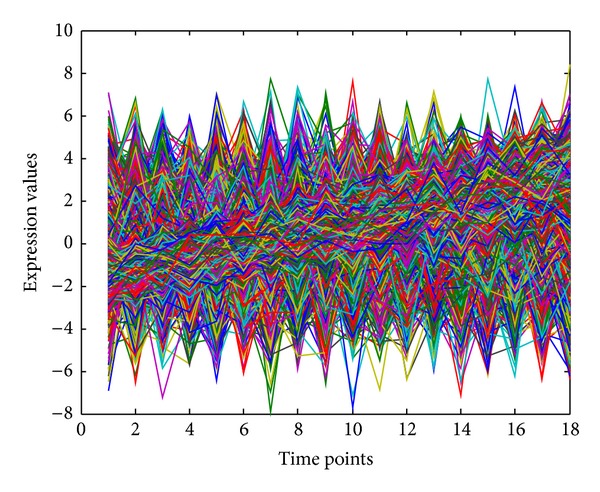
Plot of 4000 expression profiles for evaluating significance analysis method.

In this section, we use two synthetic datasets to evaluate our proposed significance analysis method and cluster analysis method, respectively. To evaluate the significance analysis method, we generate one synthetic dataset that consists of 2000 noisy gene expression profiles based on model (13) and 2000 time-course gene expression profiles based on model (1). All 4000 expression profiles are depicted in Figure 1, from which one can hardly differentiate time-course gene expression profiles from noisy ones. To measure the performance of significance analysis, we employ two widely used indices: sensitivity and specificity, which can be defined as follows [34]:

(21) Sensitivity=number  of  true  positivesnumber  of  true  positives+number  of  false  negatives,Specificity=number  of  true  negativesnumber  of  true  negatives+number  of  false  positives,

where

 true positive is a time-course gene expression profile identified as it is;
 false positive is a time-course gene expression profile identified as it is noisy;
 true negative is a noisy gene expression profile identified as it is;
 false negative is a noisy gene expression profile identified as it is time-course.

**Figure 2:**
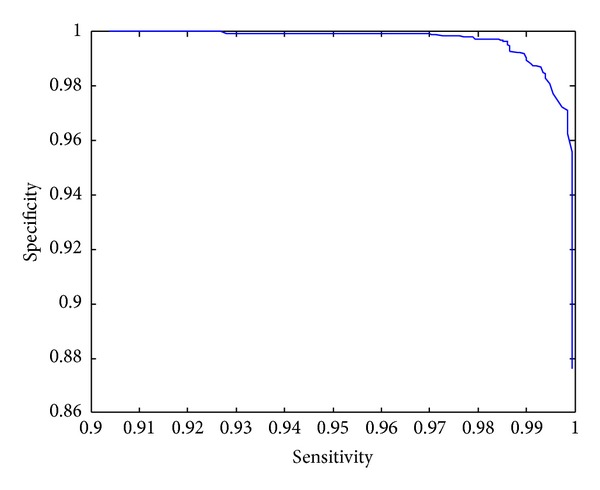
Plot of sensitivity versus specificity.

The sensitivity and the specificity depend on thresholds which determine if an expression profile is time-course or noisy. In general, the sensitivity is increasing, while the specificity is decreasing and vice versa. However, a good method is expected to have both high sensitivity and specificity.  Figure 2 depicts the curves of sensitivity versus specificity over various thresholds. From this figure, we can see that both sensitivity and specificity can be as high as of 99% for a specific threshold, which indicates that our proposed significance analysis methods are excellent.

**Figure 3:**
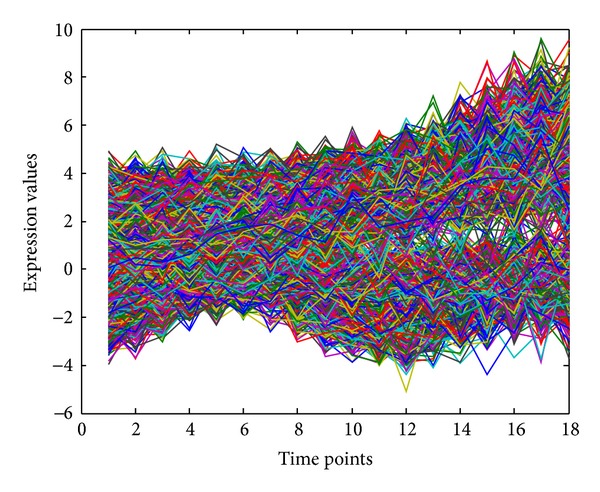
Plot of expression profiles for evaluating cluster analysis method.

To evaluate our proposed cluster analysis method, another synthetic dataset consisting of six clusters is generated from model (1), where different clusters have different randomly selected parameters with some large variances. In each cluster, all profiles are generated with model parameters for this cluster with some random perturbations. All generated profiles are plotted in Figure 3, from which one can see that all time-course gene expression profiles are mixed up. To measure the quality of clustering results, we use the adjusted Rand index (ARI) [35], which originally is to measure the degree of agreement between two partitions of the same set of objects. Given two partitions of *N* objects, the *r*-cluster partition *U* = {*u*_1_,…, *u*_*r*_} and the *s*-cluster partition *V* = {*v*_1_,…, *v*_*s*_}, the ARI is defined as follows [35]:

(22) ARI=∑i=1r∑i=1s(nij2)−1/T∑i=1r(ni.2)∑i=1s(n.j2)1/2[∑i=1r(ni.2)+∑i=1s(n.j2)]−(1/T)∑i=1r(ni.2)∑i=1s(n.j2),

where *T* is the number of pairs of *N* objects, *n*_*ij*_ is the number of objects that are both in clusters *u*_*i*_ and *v*_*j*_, *i* = 1,…, *r*, *j* = 1,…, *s*, and *n*_*i*._ is the number of objects in cluster *u*_*i*_, while *n*_.*j*_ is the number of objects in cluster *v*_*j*_. From these definitions, we have

(23) $$\begin{array}{c} T=\frac{N\left(N-1\right)}{2},\;\;n_{i.}=\overset{s}{\underset{j=1}{\sum }}n_{ij},\;\;n_{.j}=\overset{r}{\underset{i=1}{\sum }}n_{ij}. \end{array}$$

The expected value of ARI is 1 when two partitions agree perfectly and 0 when they are selected at random.

**Figure 4:**
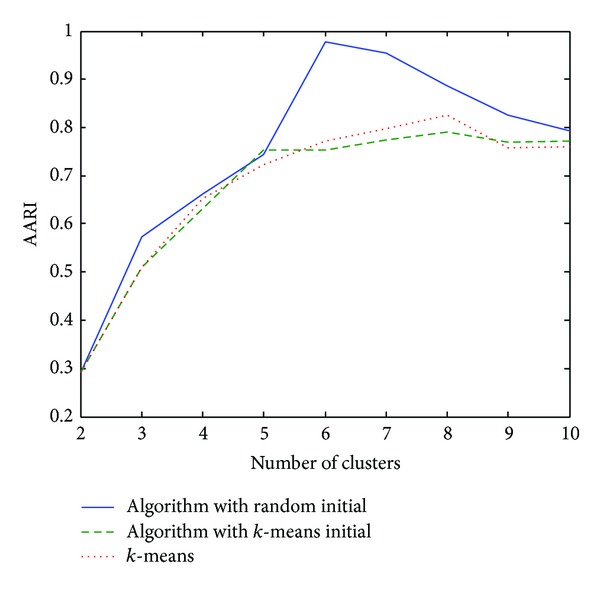
Plot of AARI with different numbers of clusters.

As the results of clustering are sensitive to the initial partition, we run our proposed clustering algorithm and competing clustering algorithms 30 times on the synthetic dataset and calculate the average ARI (AARI) for each algorithm.  Figure 4 depicts the AARI of three algorithms named “algorithm with random initial,” “algorithm with *k*-means initial,” and “*k*-means” over several different numbers of clusters, where “algorithm with random initial” means our proposed clustering algorithm with randomly chosen initial partition, “algorithm with *k*-means initial” means our proposed clustering algorithm with *k*-means result as initial partition, and “*k*-means” is an algorithm coded in the MATLAB software for *k*-means clustering method. Those values of AARI are also listed in Table 1.

From Figure 4 and Table 1, one can see that our algorithm with random chosen initial partitions outperforms the other two algorithms. Particularly, at the correct number of clusters, the ARRI from our algorithm with random chosen initial partitions reaches its maximum. The quality of our algorithm with *k*-means result as initial partitions is comparable with that of *k*-means, which indicates that after *k*-means falls in a local optimum, our algorithm cannot escape from that local optimum and thus inherits the drawbacks of *k*-means. This also suggests that our developed algorithm should combine with random chosen initial partitions.

## 4. Applications to a Real-Life Gene Expression Data

In this section, we apply our proposed significance analysis and cluster analysis method to a real-life gene expression dataset which is collected from the alpha-synchronized experiment [2]. To study the mitotic cell division cycle of yeast, Spellman et al. [2] have monitored more than 6000 genes of yeast (*Saccharomyces cerevisiae*) at 18 equally spacing time points in the alpha-synchronized experiment. The original dataset is publicly available at http://genome-www.stanford.edu. Genes with missing data are excluded in this study. The resultant dataset contains the expression profiles of 4489 genes.

**Figure 5:**
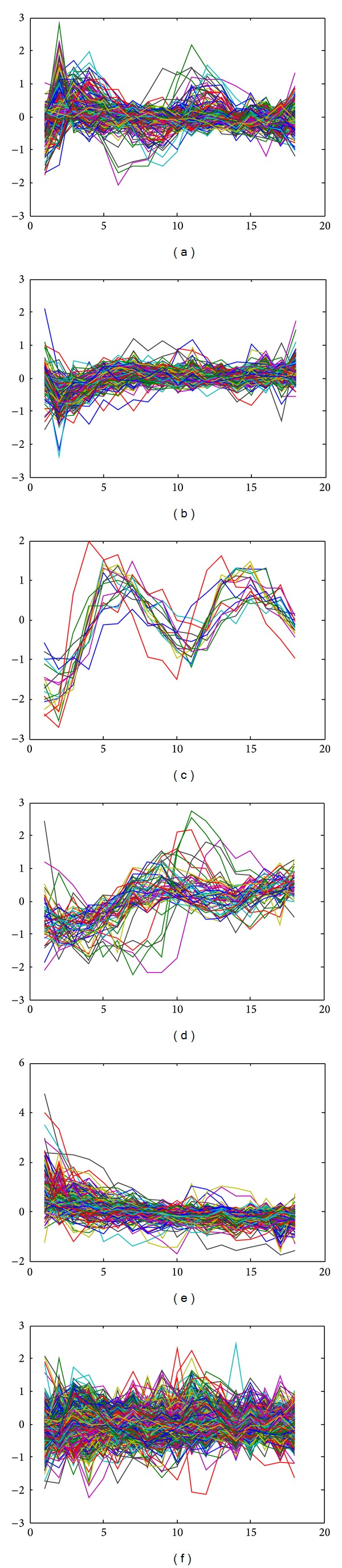
Plot of gene expression profiles. (a)–(e) show gene expression profiles for one of five clusters. (f) shows gene expression profiles which are determined as noises.

We first apply our proposed significance analysis method to this dataset and set the significance level *γ* = 0.1. As a result, 846 genes are determined to be involved in the alpha-synchronized cell division cycle process, while the other 3643 genes are determined to be noises with respect to this process.  Figure 5(f) depicts these 3643 expression profiles. From Figure 5(f), most of the expression profiles look like noises and are not related to the alpha-synchronized cell division cycle process according to the results in [2]. Then we apply our proposed clustering algorithm to the subset consisting of 846 genes involved in the alpha-synchronized cell division cycle process. According to the biological meaning of this process [2], the reasonable number of clusters is 5. The model parameters identified for each of the five clusters are listed in Table 2. From Table 2, for all clusters the values of parameter *α*_*k*_ are negative numbers, which are reasonable. As the cell division cycle is a stable biological system, after a perturbation such as the alpha synchronization, the system tends to its stable attractor. Therefore the degradation rate represented by *α*_*k*_ should be negative.

Furthermore, the values of model parameters *a*_*k*_ and *b*_*k*_ determine the importance of periodic components. From Table 2, the module of parameters *a*_*k*_ and *b*_*k*_ is the largest, while the absolute value of parameter *α*_*k*_ is small for Cluster 3. This indicates that 17 genes in Cluster 3 are periodically expressed in this process, which can be verified from Figure 5(c). Actually all 17 genes in this cluster have also been identified as periodically expressed genes in [2]. The module of parameters *a*_*k*_ and *b*_*k*_ is the second largest for Cluster 5, while the absolute value of parameter *α*_*k*_ is very large for Cluster 5. As a result, gene expression profiles in Cluster 5 are quickly degrading while hardly displaying periodicity as shown in Figure 5(e). According to the estimated values of model parameters, expression profiles in other clusters can similarly be explained.

## 5. Conclusions 

In this paper, we have presented a significance analysis method and a cluster analysis method for time-course gene expression profiles. In these methods, time-course gene expression profiles are modeled by a nonlinear model, which is a generalization of several existing models. To estimate the parameters, which is key to the developed significance analysis method and a cluster analysis method, we propose a two-step linear least squares method. One synthetic dataset has been employed to verify our developed significance analysis method in terms of sensitivity and specificity, while another synthetic dataset has been employed to verify our developed cluster analysis method in terms of AARI. The results have shown that both of our developed methods outperform some existing methods. The application to one real-life biological dataset illustrates that the analysis results of our developed methods are in agreement with the existing results. The reconstruction of gene regulatory network from time-course gene expression data is a very important issue in systems biology [36]. Obviously, noisy genes should be excluded from gene expression data for reconstructing gene regulatory networks. In the future, we may combine our method with other methods as in [36] to reconstruct gene regulatory networks.

## Figures and Tables

**Table 1:** The values of AARI for different clustering methods on synthetic data.

No. of clusters	2	3	4	5	6	7	8	9	10
Random initial	0.2915	0.5741	0.6636	0.7549	0.9787	0.9516	0.8862	0.826	0.7944
*k*-means initial	0.2915	0.4875	0.6741	0.7168	0.7732	0.7668	0.7666	0.7739	0.753
*k*-means	0.2915	0.5099	0.6352	0.7047	0.8001	0.7635	0.8189	0.7849	0.7827

**Table 2:** The model parameters for each cluster.

Parameters	Cluster 1 (315)	Cluster 2 (233)	Cluster 3 (17)	Cluster 4 (53)	Cluster 5 (228)
*α*_*k*_	−1.1543	−1.7033	−0.6612	−0.5111	−1.8483
*ω*_*k*_	9.8108	9.8673	7.1631	7.0517	8.736
*a*_*k*_	0.0234	0.2675	1.0948	0.0024	0.4427
*b*_*k*_	0.1389	0.033	−1.2261	0.1248	−0.6807
*c*_*k*_	−0.1287	0.1422	0.3353	0.5748	−0.3738
*d*_*k*_	0.1383	−0.1372	−0.2723	−0.6011	0.3946
